# Energy and Nutrient Intake Gaps and Socioeconomic Determinants of Ultra-Processed and Less-Processed Foods Consumed in Ethiopia: Evidence from National Food Consumption Survey

**DOI:** 10.3390/nu17172818

**Published:** 2025-08-29

**Authors:** Kifle Habte Balcha, Stefanie Vandevijvere, Annette van Onselen, Muthulisi Siwela, Masresha Tessema, Nqobile Monate Mkolo, Tibebu Moges, Edith J. M. Feskens, Dejen Tesfaw, Inge D. Brouwer

**Affiliations:** 1 Ethiopian Public Health Institute, Nutrition, Environmental Health and Non-Communicable Disease Research Directorate, Addis Ababa P.O. Box 1242, Ethiopia; 2 Dietetics and Human Nutrition, School of Agricultural, Earth and Environmental Sciences, University of KwaZulu-Natal, Private Bag X54001, Durban 4000, South Africasiwelam@ukzn.ac.za (M.S.); 3 Department of Epidemiology and Public Health, Scientific Institute of Public Health, Sciensano, 1050 Brussels, Belgium; stefanie.vandevijvere@sciensano.be; 4 Department of Life and Consumer Sciences, UNISA, Private Bag X6, Florida, Johannesburg 1710, South Africa; 5 Department of Biology and Environmental Science, Sefako Makgatho Health Sciences University, Ga-Rankuwa, 139, Pretoria 0204, South Africa; 6 Division of Human Nutrition and Health, Wageningen University and Research, 6708 WG Wageningen, The Netherlands; 7 Department of Statistics, College of Natural and Computational Sciences, Addis Ababa University, Addis Ababa P.O. Box 1176, Ethiopia

**Keywords:** ultra-processed foods (UPF), less-processed foods, minimally processed foods, micronutrients, NOVA foods, socioeconomic determinants, multilevel mixed-effects GLM

## Abstract

Introduction: Consumption of ultra-processed food (UPF) is associated with poor diet quality and a risk for non-communicable diseases (NCDs). This study explores the energy contribution of NOVA foods and the nutrient gaps. Methods: The study sourced data from the previous Ethiopian National Food Consumption Survey (NFCS). It covered 8254 households, 8254 women of reproductive age (15–45 years old), and 7272 children (6–45 months old). Results: The most consumed UPF in children were biscuits, cookies, soft drinks, and semi-solid palm oil; while cow and human milk, whole wheat bread, a range of legumes, tubers, and cereal-based foods were among NOVA1. In both children and women, the largest dietary energy intake was from NOVA1 (74.6% and 79.0%), processed culinary ingredients (18.3% and 14.0%), processed foods (1.9% and 3.5%), and UPF (5.1% and 3.5%), respectively. Higher intake of energy from UPF was found in urban residences, wealthier households, and women with higher education. However, NOVA1 was more dominantly consumed in rural than in urban areas. Micronutrient and macronutrient gaps were observed compared to the recommended nutrient intake (RNI). The intake of fruits and vegetables was also considerably low compared to the WHO recommendation (≥400 g/day for adults, and ≥250 g/day for children). Conclusions: Adequate intake of micronutrients, fruits, and vegetables is essential to meet the RNI and could have reduced existing body micronutrient deficiencies, such as vitamin A, zinc, iodine, calcium, vitamin D, and selenium prevalence. Whether UPF intake in urban areas is associated with insufficient availability and access to NOVA1 foods or just due to the higher provision of UPF and gained popularity needs additional investigation. Further study is recommended to simulate the impact of increased fruits and vegetables and/or reduced intake of selected UPF, salts, and oils on NCD markers or mortality in the country.

## 1. Introduction

Noncommunicable diseases (NCDs) were responsible for at least 43 million deaths globally in 2021. In 2024, 18 million people died from NCDs, younger than 70 years of age. The four groups of diseases (cardiovascular, cancers, chronic respiratory, diabetes, and kidney disease account for 80% of all premature NCD deaths. Unhealthy diets, which mainly include ultra-processed foods (UPF), are among the major risk factors for dying from an NCD [1]. Daily energy intake (exceeding 50% of total energy) [2,3] from UPF is directly linked to a higher consumption of added sugar, sodium, total fat, and saturated fat. Essentially, UPF is contrary to the Mediterranean diet and diet quality (level of minerals, vitamins, fiber, carbohydrate) contents, which also signals unhealthy diet consumption in the population [1,4,5]. Food categorization, like the NOVA classification, has been used when estimating energy intake from UPF. It divides foods into four categories according to their processing level [6]. Table 1 indicates the NOVA food definition and classifications. However, more detailed classification and examples of NOVA food categorization can be obtained in [7,8].

Based on current scientific evidence, UPF supply is lower in low and middle-income countries (LMICs), including in Africa, compared to high-income countries (HICs). However, the supply rate in LMICs is faster than in HICs, as described in [11]. For instance, there has been a rise in per capita UPF sales in Nigeria and South Africa from 2004 to 2018 [12]. However, studies regarding the national energy consumption from UPF, less-processed foods, or their supply are scarce in Ethiopia. According to a secondary data analysis by the National Information Platform for Nutrition (NIPN) in Ethiopia, per capita daily calorie intake from sugar-sweetened beverages and sugar-sweetened snacks in 2010 was (4.4 and 3.7), but in 2016 it was (7.2 and 3.1), respectively [13]. Furthermore, based on the Ethiopian Food Systems Dashboard report [14], the availability of fruits and vegetables in Ethiopia was poor compared to the world and East African countries, but total per capita UPF sales were also very low [15]. According to evidence from systematic reviews of observational studies, consumption of UPF, particularly, higher daily energy intake from UPF is associated with the risk of diet-related NCDs, such as increased overweight and obesity (body mass index: BMI ≥ 25 kg/m^2^) and (BMI ≥ 30 kg/m^2^, respectively), high blood pressure (Systolic/diastolic 140/90 mmHg), metabolic syndromes (central obesity, high blood sugar, and triglycerides levels, low-density lipoprotein cholesterol (LDL-c) and lower low level of high-density lipoprotein cholesterol (HDL-c) [1,3,4,16,17]. Moreover, it is associated with the risk of irritable bowel syndrome, depression, cerebrovascular diseases, cancer, and all-cause mortality [18,19]. However, except for some studies available, such as [20], most of the adverse effects of UPF associated with various forms of non-communicable diseases, including overweight/obesity, and beyond, have been reported based on longitudinal and cohort studies. It is challenging to design clinical trials on the effect of UPF on human health due to ethical sensitivity. But, people are consuming UPF due to various reasons, for instance, due to sensory appeal: taste and palatability [21,22,23,24], due to relatively lower price, and cooking convenience [21,25,26], because of excessive media advertisements and peer pressure [23,26], and due to the addictive nature of UPFs, has been mentioned [21,22].

There is a scarcity of reports in Ethiopia on UPF intake and association with NCD. However, there have been various reports on the prevalence of NCD. According to a study by [27], the incidence rates, death rates, and disability-adjusted life years (DALY) rates of NCD in Ethiopia in 2019 were 190,000 (180,000–200,000), 550 (500–600), and 12,200 (10,400–14,200) per 100,000 population, respectively. NCD-associated deaths were higher in Addis Ababa city (55% of the total deaths), compared to other regions (51% in Tigray, 45% in Harari, 43% in Amhara, and 42% in Dire Dawa). From 2010 to 2019, the rise in the percentage of deaths was 21% (8–35%), the rise in incidence rate was 25% (24–26%), and the rise in DALYs was 17% (6–29%). However, the NCD determinants, such as UPF, and less processed food intake, and their association with socioeconomic factors have not been investigated in the country. In addition, the extent to which the national dietary intake has a gap compared to micro and macronutrient recommended nutrient intake (RNI) or recommended daily allowance (RDA) has not been reported using intake data. Therefore, this study aimed to examine the level of energy contributed by each NOVA food group and to investigate whether the consumption had fulfilled the micro and macronutrient daily requirements based on RNI in Ethiopia.

## 2. Materials and Methods

### 2.1. Survey Data Collection

**Figure 1:**
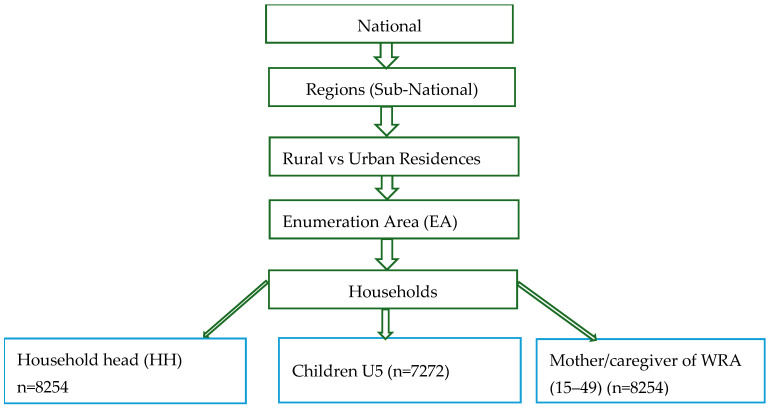
Survey sampling frame.

Data for this analysis were obtained from the Ethiopian National Food Consumption Survey (NFCS). The survey was designed based on a random sampling of the Ethiopian population using a two-stage stratified cluster sampling procedure to select households. In the first stage, 324 enumeration areas (EAs) were randomly chosen using probability-proportional-to-size (PPS) allocation of EAs found in the 11 sub-nationals (Regions). In the second stage, 26 eligible households were selected within each EA (clusters) using systematic random sampling to pick eligible women of reproductive age group (WRA) aged 15–45 years old, and under-five children in the households. The survey was conducted in the lean season (June to September 2011). Finally, the survey included 8254 households, 8254 WRA, and 7272 children aged 6–45 months. The survey was composed of dietary intake, anthropometry, and socio-demographic data. Information was taken from the household head, mothers/caregivers, and children. The data contains dietary intake, household socioeconomic status (SES), mother’s or caregiver’s age, literacy level, mother’s job, anthropometry (BMI), and living residence (urban/rural). Figure 1 below indicates the sampling framework followed during the survey. The residence categorization was carried out based on existing Ethiopian Statistical Service (ESS) criteria for urban and rural residences [28].

### 2.2. Dietary Assessment and Food Classification

A quantitative 24 h dietary recall (24HDR), and a multiple-pass 24HDR collection method was used to improve the recall of a variety of foods, portion size, and ingredients consumed, as explained in Gibson and Ferguson [29], also described in [30]. From Pass 1 to Pass 3, a systematic track of participants’ 24 h dietary intake, including types of food and drink, portion sizes, and recipe details, is then finally checked for data accuracy and completeness in Pass 4. There is a 24HDR validation study reported in the Ethiopian population [31]. To estimate the portion size, tools such as direct weight measurement, standard unit sizes, and proxy weight estimations like play dough, water, rice, maize flour, cups, and glasses, which support accurate estimation, were used, and dietary collection formats were standardized. Data collectors were trained for about 10 days, and a pilot survey was conducted before running the actual survey. The dietary tools were rechecked for their validity, accuracy, and precision after the pilot study. Locally known language translators were used in different regional parts of Ethiopia during the collection whenever necessary. Leftover foods were considered in the calculation, and the nutrient compositions of each food were estimated using the Ethiopian Food Composition Table (EFCT) [32]. Energy and macronutrients such as total protein, carbohydrate, fat, fiber, phytate, and micronutrients like zinc, calcium, iron, vitamin A, vitamin C, thiamin, riboflavin, and niacin intake were estimated. To estimate the daily nutrient requirements, age-based RDA and RNI cut-off values were decided according to the World Health Organization & Food and Agriculture Organization, and the Institute of Medicine (IOM) reports [33,34,35,36,37,38]. For NOVA-based analysis, all consumed unique ingredients and their label name were sorted before assigning ingredients to one of the four NOVA food classifications. Finally, a total of 792 unique ingredients were identified and categorized into one of the four NOVA food groups based on the classification described in Table 1. The file was saved in an Excel spreadsheet and then imported into Stata version 18 (College Station, TX, USA). The unique ingredient-containing file was merged into the original large file. Therefore, the merging of data enabled categorization of the whole original ingredient-based data into either NOVA1, NOVA2, NOVA3, or NOVA4, according to the NOVA food classification used in [39]. Energy contributed by each NOVA food was estimated by summing up the energy that came from ingredients of each of the NOVA categories, divided by the sum of total NOVA foods, and multiplied by 100. A detailed example of Ethiopian food items classified according to the NOVA food system is shown in the Supplementary Materials.

### 2.3. Anthropometric Measurement and Categorization

The body weight of survey participants was measured on a Seca 874 digital weight scale to the nearest 0.1 kg without heavy clothing, and barefoot. Standing height was measured on a wooden height measuring board to the nearest 0.5 cm with light clothing and bare feet. For children less than 2 years, a recumbent length was taken. Height and weight measurements were recorded in duplicate but triplicated if there was a large disagreement between the first and second measurements. BMI was calculated, weight in kilograms divided by height in meters squared, and classified as underweight (BMI < 18.5), normal (BMI 18.5–24.9), overweight (BMI 25.0–29.9), and obese (BMI ≥ 30) for WRA [40]. However, children’s anthropometric data were measured based on the BMI-for-age Z-score (BAZ). Later, BAZ was categorized into underweight when BAZ < −2 SD from the median of the reference population, normal weight (−2 SD < BAZ < 2 SD) from the median of the reference population, and overweight and obese was categorized when BAZ > 2 SD above the median of the reference population according to the method used in [41].

### 2.4. Household Socioeconomic Status (SES)

The data was collected using well-structured questionnaires that the household head had to respond to: whether owning a house, and the type of the house such as flooring, wall, and roofing; owning agricultural land, any type of businesses running, owning livestock, access to electricity, access to water, source of water, types of toilet facility, availability of vehicles, such as cars, motorbike, bicycle; availability of home utilities such as refrigerator, computer, television, radio, cell phones, electric mitad/grills and stoves, also employment status. The data was collected based on a dummy variable “Yes = 1” and “No = 0” responses. The wealth index of the household was calculated based on the response for available or unavailable household assets during the survey. The responses were later combined into a single wealth index factor using principal component analysis (PCA, factor analysis). The scale was finally ranked, after being subdivided into five equal strata called “wealth quintiles” according to the method mentioned [42]. That means, 1 = “Poorest”, 2 = “Poor”, 3 = “Middle”, 4 = “Rich”, 5 = “Richest”. This quintile-based data was initially constructed by the former Ethiopian Central Statistics Agency (CSA) and was available in the secondary data. But the quintile stratum was shrunk into three parts (Terciles Wealth Index) for this analysis purpose: Poorest (1) = (Poorest, and Poor), ‘Middle’ (2) = Middle, and Richest (3) = (Rich, and Richest). This tertile wealth index categorization is shown to enhance the model’s fitness while fitting the dependent and all the independent variables.

### 2.5. Statistical Analysis

According to the Ethiopian Central Statistical Agency (CSA), a regional distribution of no more than 40 clusters in the largest regions and no less than 20 in the smaller regions was priorly estimated, with a total of 324 enumeration areas (EAs) in the country, and an average of 26 households per EA estimated. Finally, a total of 8254 HHs, 8254 WRA, and 7272 children aged 6–45 months were included in the survey. The energy data, particularly energy consumed from UPF, did not conform to the Gaussian normality assumption. Also, since it was consumed relatively less, some of the NOVA4 data had zero values; thus, we have transformed each NOVA energy data to estimate the share of energy from each NOVA food. Also, since the variability of energy, macro, and micronutrient, salt, sugar, and edible oil intake was extremely high; therefore, 25%, 50%, 75%, and 95% of the data were presented in the descriptive tables. To check whether an association exists between energy sourced from UPF or minimally processed foods with sociodemographic variables, a multilevel mixed-effects generalized linear model (meglm) with random and fixed (main effect) variables was used. One of the important assumptions of a meglm model is that the dependent variable should be continuous, positive, and it can accommodate right-skewed data. This model was preferred because it also gives options to consider a random variability, in our context, due to the survey hierarchy nature (National to Regions) put first in the model, then a clustering effect (enumeration area, EA) next. The sociodemographic factors like residence, education level, family wealth status, weight (BMI), mother’s job, etc., were put as fixed variables. In addition, in the model we included the gamma family and log link function, which is suitable for continuous positively skewed dependent variables with multiple independent variables, each having two or more levels. The general model and exponential form of the model with dependent variable (UPF intake) and the explanatory variables in WRA and children are shown in Equations (1), (2) and (3), respectively.

Model equation with long link function, combined with random and fixed effects:

log(**μ**ijk) = β_0_ + β_1_X_1_ijk + β_2_X_2_ijk + … + β_n_X_n_ijk + b_0_k (1)

Exponential form of the model structure with variables:

**μ**(vjk) = exp(β_0_ + β_1_xBMI (vjk) + β_2_xSocioeconomic Status (vjk) + β_3_xMother’s Education (vjk) + β_4_xResidence (vjk) + β_5_xmother’s Age (vjk) + uk) (2)

**μ**(vjk) = exp(β_0_ + β_1_xResidence (ijk) + β_2_xChild Age (ijk) + β_3_xChild BMI (ijk) + β_4_xSocioeconomic Status (ijk) + β_5_xMothe’s Education (ijk) + β_6_xMother’s Job (ijk) + uk + vjk) (3)

where

µ: the mean UPF energy intake of an individual

uk: random intercept for the region

vjk: the random intercept for clusters within the regions

X_1_, X_2_ … X_n_: the explanatory variables of interest

β0: global intercept

Beta: β_1_ to β_n_, β_1_ to β_5_, and β_1_ to β_6_ are coefficients of fixed effect variables in (1), (2), and (3), respectively.

In the meglm model, energy from UPF (NOVA4) and energy from minimally or less-processed foods (NOVA1) were taken as response variables independently. Other socioeconomic determinants mentioned above were taken as explanatory variables. Those individuals who had missing sociodemographic data were not included in the regression model. Statistical significance was considered at *p* < 0.05 and 95% CI after Holm–Bonferroni correction. Model fitness for meglm was checked, based on Akaike Information Criterion (AIC) = 47,291.8 and Bayesian Information Criterion (BIC) = 47,291.6, as compared to the multilevel mixed-effects (xtmixed) model with AIC = 94,721.8 and BIC = 94,853.6 for UPF intake in WRA. For children, AIC = 44,832.2 and BIC = 44,956.3 in meglm, and AIC = 84,773.9 and BIC = 84,898.0 in the xtmixed model. Thus, in our case, the meglm was preferred with lower AIC and BIC values than its counterpart xtmixed model.

## 3. Results

Most of the survey participants (>70%) in both WRA and children were from rural parts of Ethiopia. For WRA, the dominant age was early adulthood (22–34) years old, while 1–3 years old were the dominant age (42.7%) for children. Nearly two-thirds (63.4%) of WRA had normal body weight (BMI between 18.5 and 24.9), followed by underweight (27.3%), and the rest (9.3%) were overweight/obese (BMI ≥ 25). About two-thirds had taken no formal education (62.7%), followed by primary school (1–8 grade) (23.8%). The majority of WRA (45%) were serving as housewives, whereas 28.7% were formally employed, 13.4% were part-time or working irregularly, and the rest were unpaid family workers/supporters. Based on household wealth terciles, 39.6% were the poorest, and 40.7% were the wealthiest households. Detailed information about the socio-demographic characteristics of participants is shown in Table 2 below.

Most of the WRA and their children did not consume fruits per 24HDR; those consumed were just 5% for children with a Median of 0.0 g (0.0, 0.0) and about a third (1/3) for mothers in the 24HDR period with a median of 0.0 g (0.0, 3.4). However, those who do not consume vegetables were 39% of children and just a quarter of the WRA. Median vegetable consumption in grams per day for WRA was 61.8 g (1.78, 207.72), whereas 7.8 g (0.0, 54.20) for children. Those who consumed fruit once, twice, or three times per day in WRA were (12.7%, 12.6%, 9.0%), while (4.1%, 1.1%, 0.5%) for children, respectively (Table 3). Nearly 66.0% of WRA and 95.0% of children did not consume any fruits in the last 24HDR. As many varieties of NOVA1 food ingredients were consumed, they were categorized into cereal-based, meat and meat products, milk, egg, fruits, vegetables, spices, herbs, roots, and tubers. However, the rest of the NOVA ingredients were presented without categorization into ranges of food groupings (Supplementary Materials). The total average per day energy intake in WRA (n = 8254) was 1780 kcal ± 987, with median and quartiles of 1587.0 kcal (1095.9, 2254.9), whereas the total average energy intake in children (n = 7272) was 669.6 kcal ± 513.7, with a median and quartile of 553.0 kcal (298.0, 912.8).

In both children and women, the largest dietary share of energy was from minimally processed foods: 74.6% (95% CI; 74.2–75.1) and 79.0% (95% CI; 78.6–79.4); from processed culinary ingredients: 18.3% (95% CI; 17.9–18.8) and 14.0% (95% CI; 13.7–14.3); from processed foods: 1.9% (95% CI; 1.7–2.0) and 3.5% (95% CI; 3.3–3.8), and from UPF: 5.1% (95% CI; 4.9–5.4) and 3.5% at 95% CI (3.3–3.7), respectively. The most consumed UPF by children were biscuits, cookies, soft drinks, and semi-solid palm oil; while fresh cow milk, human milk, whole wheat bread, legumes, roots and tubers, and a range of homemade cereals and cereal-based foods were consumed as minimally processed (NOVA1) foods.

Energy and macronutrients (protein, carbohydrates, and fat) and micronutrients such as zinc, calcium, iron, vitamin A, vitamin C, niacin, thiamin, phytate (Phy) contribution by a variety of NOVA foods consumed are shown below in Table 4 for WRA and Table 5 for children. Large portion of the total energy consumed in WRA and children was contributed by carbohydrates 1136.5 kcal (284.1 g) and 364.7 kcal (91.2 g) followed by fat, 211.9 kcal (23.5 g) and 96.2 kcal (10.7 g), and then followed by protein 150.7 kcal (37.7 g), and 54.7 kcal (13.7 g), respectively (Table 4 and Table 5). The median daily salt consumption in WRA was above one teaspoon (Table 4). The median of total Phy:Fe and Phy:Zn molar ratio was 3.1 and 18.0 for women, whereas 2.3 and 15.3 for children, respectively (Table 4 and Table 5).

Median daily intake of vitamin A, thiamine, riboflavin, niacin, vitamin C, calcium, iron and zinc was 32.7 µg, 1.2 mg, 0.9 mg, 8.0 mg, 22.8 mg, 347.0 mg, 38.0 mg and 6.6 mg for WRA participants; whereas, 24.4 µg, 0.4 mg, 0.4 mg, 2.7 mg, 8.0 mg, 152.6 mg, 9.9 mg and 2.0 mg for children, respectively (Table 4 and Table 5). The median total Phy:Ca and [Phy × Ca]/Zn molar ratio was 0.4 and 148.8 for women, but 0.6 and 59.4 for children, respectively. Table 6 below indicates the calorie, daily nutrient intake, and % RNI achieved in both WRA and children. Except for micronutrients such as iron and thiamine RNI in children (1 to 4 years old) and WRA, and protein in children (1 to 4 years old), the intake has shown a gap in fulfilling most of the macro and micronutrients.

According to Table 7 below, the consumption of energy from UPF and the percent contribution to daily consumption was relatively minimal. However, the % contribution of UPF was comparably higher in Addis Ababa and Harari regions, followed by Dire Dawa for both children and WRA. In contrast, the least contribution of UPF for daily energy was observed in the Afar region for children and the Gambela region for WRA. Table 8 below displays the energy consumption from UPF and its association with the sociodemographic characteristics of the children.

There was a significant difference between the child’s residence, age, BMI, SES, mother’s education level, and the mother’s job who served as a housewife (Table 8). Those children living in urban areas had more than double the exposure (OR: 2.55, 95% CI; 1.66–3.84) to energy intake from UPF compared to those living in rural areas. Regarding age, those children above 2 years old generally consumed more energy from UPF compared to 6–12-month-old children. Children above 3 years old were the most consumer age group (OR: 6.75, 95% CI: 3.42–13.84) compared to the reference age (6–12 months). However, there was no significant difference between overweight/obese (BMI for age z-score, BAZ > 2 SD) children in the consumption of energy from UPF compared to those underweight (BAZ < −2 SD) children. Children from the richest and middle-income households did not significantly consume high energy from UPF after adjustment for Holm-Bonferroni correction. However, children whose mothers attended primary school (G: 1–8), high school (G: 9–12) and tertiary level (G: >12) had high chance of taking energy from UPF (OR: 1.51, 95%: 1.35, 1.70), (OR: 1.77, 95% CI; 1.50, 2.10) and (OR: 1.47, 95% CI; 1.12, 1.93), respectively, compared to children whose mothers not attended formal education. Also, children took less energy from UPF of those whose mothers served as housewives compared to mothers who partook in formal jobs (Table 8). In contrast, no significant difference was found between children’s residences, nor in their BMI category for energy intake from less-processed foods (NOVA1). However, a significant difference was observed among children’s age, SES, mother’s education level, and mother’s job involvement. That means, all other age groups consumed more than the reference groups (6–12 months), but the highest consumer of energy from NOVA1 (OR: 2.43, 95% CI; 2.32–2.55) was 2–3-year-old children (Table 9). Wealth index-based analysis indicated that in contrast to the richest households, intake of energy from NOVA1 was significantly higher for those children from the poorest and middle-income families (OR: 1.23, 95% CI; 1.16–1.31), and (OR: 1.14, 95% CI; 1.07, 1.21), respectively. Conversely, children whose mothers attended high school and tertiary schools had shown greater consumption of energy from NOVA1 (OR: 1.16, 95% CI; 1.08–1.25) and (OR: 1.29, 95% CI; 1.15–1.45) compared to children whose mothers attended no formal education.

Table 10 and Table 11 below demonstrate the association of UPF and minimally processed foods (NOVA1) consumption with their sociodemographic determinants in WRA. In brief, living residence, SES, and mother’s education have significant associations with the intake of energy from UPF. Those residing in urban areas compared to rural areas were likely exposed to higher levels (OR: 3.77, 95% CI; 2.40–5.92) of energy intake from UPF. A rise in energy intake from UPF was observed in the richest households in contrast to the poorest ones (OR: 39%, 95% CI; 20–63%). Conversely, those WRA who attained elementary school, high school, and tertiary level had higher (OR: 38%, 95%CI; 24–53%), (OR: 66%, 95%CI; 42–94%), and (OR: 41%, 95%CI; 12–78%) intake of energy from UPF, respectively, compared to those who attended no formal education. However, a significant difference was not found in the mother’s age category, BMI, and jobs involved in energy intake from UPF (Table 10). On the other hand, there was a significant difference observed in mothers’ energy consumption from NOVA1 in their residence, mothers’ age, and mothers’ job after correction with Holm–Bonferroni. Mothers who lived in urban areas had a reduction in energy intake from NOVA1 (OR: 0.80, 95%CI; 0.74, 0.86) compared to rural living mothers (Table 9). Furthermore, Teen/young-adult WRA (15–21 years old) had a higher chance of (OR: 6%, 95%CI; 2–11%) taking energy from NOVA1 foods than early-to-late middle-age groups (35–45 years old) (Table 11).

## 4. Discussion

Daily intake of more energy from NOVA1 foods has a key role in terms of ensuring the nutritional quality of foods (NOVA4 < NOVA3 < NOVA1), also minimizing the burden of diet-related NCDs in the population [44,45,46,47,48]. Compared to the energy sourced from NOVA1 foods, the share of UPF was lower in Ethiopia, which is advantageous. Our study findings showed that less-processed foods contributed the majority of NOVA food energy share (75%) for children and 79% for WRA, whereas the share of UPF was comparably minimal, 5.1% and 3.5%, respectively. Because most of the Ethiopian population (nearly 84%) lives in rural areas with arable land [49], most farmers and rural residents will likely consume their agricultural produce. Not only is ubiquitous access to UPF viable in an urban part of Ethiopia, but job-based salaries, cash incomes, and various business sources are common in the capital city and large towns. People with higher cash incomes tend to consume more UPF than those with lower incomes in Ethiopia [50]. This nature could influence and contribute to the current national lower share of energy from UPF, as most of the Ethiopian population resides in rural parts where sources of pocket money and cash incomes are less. Socioeconomic analysis of energy intake has shown that households with higher wealth income are likely to consume energy from UPF in WRA, but this was not significant for children after adjusting for the Holm–Bonferroni correction. The finding highlights that the economic power to purchase a range of available UPF might influence purchase behavior and intake. The finding coincides with the previous report in South Africa [51] and in Kenya [52], in which individuals of higher wealth index were associated with either eating more fast foods, fried, and takeaway foods or taking higher energy from UPF than lower wealth index. It also corresponds to study findings in LMICs, for instance, in Brazil [53,54], Argentina [55], Colombia [56], and India [57], where consumption, expenditure, and energy from UPF were higher in higher-income households and individuals compared to those earning less. However, these associations were not always the case; there are also scenarios in which contradictory findings exist, especially reports from developed countries, such as Australia [58], UK [59], and USA [60], where consumption of higher energy from UPF was higher in lower-income households. On the other hand, women and children living in urban parts of Ethiopia had significantly higher intake from UPF compared to those living in rural settings. This finding harmonizes with the report made by [51] and [61], in which consumption of UPF was more popular in urban areas than in rural areas. Also, a report by [62] indicated that consumption of soft drinks, sweets, and snacks was more prevalent in urban areas than in rural areas. Moreover, a study conducted by [63] showed that students who resided in urban areas were more likely to consume soft drinks daily than those in rural areas.

Mothers’ education was one of the determinants significantly associated with energy intake from UPF. Mothers who had attended elementary, high school, and tertiary level education consumed more energy from UPF than those mothers with no formal education. This result is comparable to a study report in Ethiopia by [50], as well as by [64]; also existing findings in Brazil [65,66,67], Portugal [68], Saudi Arabia [69], and Australia [58], where people with fewer years of education had less gram/energy consumption from UPF or a lower percentage share of them compared to those receiving longer education. However, education level was not significantly associated with energy intake from NOVA1 by WRA. The intake of NOVA1 foods was lower in children from higher-income families compared to those from low-income families, but this difference was not statistically significant in WRA. Conversely, the total daily energy and nutrient intake reported in this study was comparably lower than the nutrients and energy estimated in previous studies. For instance, studies conducted by [70] used crop and livestock production data, a study by [71] used Household Expenditure Survey (HES) data, and a study conducted by [72] used FAO food balance sheets data. Also, compared to a study by [73] estimated the usual intake of vitamin A, iron, and zinc. Disparities in energy and nutrient report differences could be, first, because the previous studies reported nutrients and energy based on supply and expenditure data; second, due to seasonality dynamics: as the lean season energy and nutrient intake (current study) will not be as high as harvest season intake in the country; this seasonality difference was reported by [74] using dietary intake data, also estimated by [75] using HES data. Third, we used the population median value instead of the mean, since the energy and nutrient intake data were skewed, and the median of energy and nutrients is comparably lower than the mean distribution. The percentage of RNI fulfilled for the macro and micronutrients of the food consumed (NOVA total) was estimated. It fulfilled daily iron and thiamine requirements for children (1 to 4 years old) and WRA, also daily carbohydrate requirements for WRA, and protein requirements for children (1 to 4 years old). However, a gap still exists to meet the RNI of nutrients such as vitamins and minerals presented in this study, which corresponds with gaps in the intake of animal-source foods (milk, egg, meat, and fish) reported by [41,76,77]. Also, lower intake of vegetables (median 61.8g and 7.8g in WRA and children), and zero median intakes of fruits were conformed with previous reports in Ethiopia [78,79,80]. According to the WHO 2023 recommendation, intake of fruits and vegetables for adults (WRA) ≥ 400 g/day and for under-five children (≥250 g/day), respectively [43]. These dietary intake gaps have been reflected in biomarkers of nutrients assessed previously and deficiency of micronutrients reported, especially for serum zinc, Vitamin A, calcium, vitamin D, selenium, and urinary iodine in children and WRA in Ethiopia [81,82,83,84,85].

In another scenario, consumption of a larger share of energy and nutrients from NOVA1 foods, in this case mainly sourced from cereals, legume-based diets, tubers, and roots, has been looked at nationally, where the bioavailability of nutrients might be one of the concerns in nutrient absorption. The phytate-to-mineral ratio in this study was presented in Table 4 and Table 5. A critical value indicative of lower bioavailability of iron in the presence of phytate in a molar ratio (Phy: Fe) is > 1.0; a ratio greater than this value suggests poor absorption of iron in the intestinal mucosa [86], and the phytate to zinc molar ratio (Phy: Zn) > 15.0 is linked to low zinc bioavailability [87]. However, a phytate to calcium molar ratio (Phy: Ca) > 0.24, and both effects of phytate and calcium on zinc molar ratio [Phy × Ca]/Zn > 200 are the cut-off value for phytate alone on calcium and (phytate plus calcium) on Zn bioavailability, respectively [88]. The median molar ratio of (Phy: Fe) in WRA and children, and (Phy: Zn) in WRA in this study were above the cut-off, while (Phy: Zn) in children was borderline high, 15.3 (7.3, 25.8). However, the median values of (Phy: Ca) and [Phy × Ca]/Zinc) were within the normal range: 0.2 (0.1, 0.3) and 142.8 (61.5, 338.2) for WRA versus 0.2 (0.1, 0.3) and 40.5 (11.8, 109.7) for children, respectively. Though dietary phytate has the potential to hinder the bioavailability of micronutrients, according to a study such as by [89], it has many protective roles, for instance, anticancer, antioxidant, and anti-inflammatory properties, preventing the risk of type 2 diabetes mellitus, the ability to prevent Parkinson’s disease, and neuroprotective effects. Furthermore, dietary phytate eradicates bone mass loss, reduces pathological calcification in organs and vascular tissues, inhibits browning, and reduces proliferation of pathogenic microorganisms in the food industry [39]. The fact that dietary phytate has both pros and cons in the human body in terms of its disease protective advantages, but it has the potential to hamper body nutrient absorption. Thus, a commendable approach could be optimization or balancing the intake of phytate-rich plant foods mixed with animal source foods; otherwise, taking multiple supplements on and off will be important, especially when following vegan and vegetarian dietary habits.

## 5. Limitations and Strengths

This study has several strengths and limitations to consider when using and interpreting the findings. First, the survey was bound to include women of reproductive age group (15–49 years) and children (6 to 45 months) only. Data was not collected particularly for men; this might limit the generalizability of the findings for the entire population group and gender in the country. However, a huge and nationally representative dietary intake sample size taken from the whole regions, households, and residence categories for the WRA and under-five children population makes it worthwhile to report and statistically powerful, can indicate what is generally looks like the dietary intake and the gaps that remain to make up in the country. Dietary assessment in the survey was conducted based on a single quantitative 24HDR. The data does not capture intra-person day-to-day variation in intake, and the variability of dietary intake would have been better accounted for by multiple 24HDR. However, during the survey data collection, to accommodate the day-to-day variations in the intake in 24HDR at the population level, data was collected by equally distributing it over the seven days of the week. Furthermore, due to the large sample size, as well as the complex nature of quantitative dietary data collection, repeated 24HDR was demanding and not feasible at the survey time. The survey was conducted at the lean season in Ethiopia, so by its nature, this might limit the availability of different foods in the HHs and the number of individuals consuming a variety of foods, frequency, and/or the portion size consumed are likely low compared to non-lean seasons. Therefore, it is very important to take this into consideration, as these findings can indicate a very low dietary intake scenario. Also, since the data covers one season, it is insufficient to show seasonal variations. This national dietary intake survey was conducted in 2011 and was reported in 2013. Since then, there might have been an evolution in NOVA food consumption patterns and nutrient intake in the population, either due to shifts in the food environment or dynamics in the socioeconomic factors, or both. Therefore, it is worthwhile to analyze and report current results or make a trend dietary intake analysis.

## 6. Implications of the Study to UPF Intake

We have briefly explained the adverse health effects of UPF reported globally in the introduction. No studies have yet investigated whether the regular consumption of UPF could have health benefits. However, the authors want to remain clear that there are circumstances in which UPF has been used, and may be used in the future, such as in places and conditions where less-processed foods cannot be obtained, during long-haul travel, which necessitates extending the shelf life of foods for many-days and months, and during necessary food fortification in appropriately selected food vehicles. Moreover, UPF may be used sparingly during life-saving events, such as consuming various energy drinks, quick energy foods, and others, during sickness, hospitalization, or other extreme physiological needs. However, as defined and explained in the first section of this study, we considered that continuous and daily high-energy intake from UPF, for instance, greater than 50% of the daily energy, could significantly affect human health, particularly by increasing overweight, obesity, diabetes, hypertension, metabolic syndromes, and other NCDs.

## 7. Conclusions

Consumption of less processed or minimally processed foods is dominant in rural Ethiopia compared to urban areas, but UPF consumption is higher in urban areas. NOVA1 foods were the major contributor to daily Ethiopian food consumption, and therefore daily energy. They provided a large share of macronutrients, such as carbohydrates and protein, also sufficiently meeting the daily micronutrient requirements, such as thiamine and iron. However, as part of NOVA1 foods, the consumption of fruits and vegetables was marginal in Ethiopia. Furthermore, there is a large gap in dietary intake in terms of meeting the daily requirements for micronutrients, such as vitamin A, riboflavin, niacin, vitamin C, zinc, and calcium. Given the low dietary adequacy and various micronutrient deficiencies in the country, optimal intake of fruits, vegetables, and animal-source foods is essential to meet daily vitamin and mineral requirements and balance the low bioavailability of plant-based diets. Whether high UPF intake in urban areas is associated with insufficient NOVA1 food availability and access, or due to the higher provision of ultra-processed foods and gained popularity compared to rural areas, warrants further study. In connection with our findings, we recommend further study on modeling the impact of increased fruit and vegetable intake and/or reduced intake of selected UPF, salts, and oils on NCD-related markers or mortality in the country.

## Figures and Tables

**Table 1:** NOVA food definition and classifications.

	NOVA1	NOVA2	NOVA3	NOVA4
Other names of NOVA foods	Unprocessed, minimally or less-processed foods	Processed culinary ingredients	Processed foods	Ultra-processed foods (UPF)
Definition of NOVA foods	Foods directly obtained from natural sources [9,10]. They are minimally processed or unprocessed foods; either consumed directly from the natural source or by removal of inedible/unwanted parts with or without the process of drying, powdering, squeezing, crushing, grinding, fractioning, steaming, poaching, boiling, roasting, pasteurization, chilling, freezing, vacuum packaging, non-alcoholic fermentation, etc., processes not adding salt, sugar, oils/fats.	Food ingredients that are not consumed alone but added as culinary ingredients.	For food products manufactured by industry, essentially, they essentially add salt, sugar, or other substances to processed foods to make them stable and more palatable.	Formulations or ingredients are typically created by a series of industrial techniques and processes.
Foods as an example	Fruit, seeds, leaves, stems, roots, tubers or animals (muscle, fat, offal, eggs, and milk); fungi, algae, and water after separation from nature [11].	Oil, butter, lard, sugar, salt, spice, and herbs that used as culinary	Bottles of vegetables or legumes (pulses) preserved in brine and vinegar, fruits, syrups, meat products, and canned fish, smoked fish, freshly baked bread, and homemade cheeses with added salt [10].	Sweets, fatty or salty packaged snacks, confectionaries and candies, packaged bread and buns, pastries, cookies, chips, biscuits, fish and poultry nuggets, carbonated and sugary drinks, margarines, other spreads, breakfast cereals, energy drinks, pizza, sausages, burgers, hot dogs, instant soups, baby formula, noodles, and desserts [10].

**Table 2:** Socio-demographic characteristics of women (15–49 years) and children (6–45 months).

Variable	Women (15–49 Years)			Children (6–45 Months)		
	Level	Frequency	(%)	Level	Frequency	%
Residence	Urban	2142	28.1	Urban	1945	26.8
	Rural	5492	71.9	Rural	5327	73.2
Age	Teen/young adult (15–21)	1296	17.0	6–12 months	1804	24.8
	Early adulthood (22–34)	4982	65.3	13–24 months	3102	42.66
	Early–late middle age (35–45)	1356	17.8	25–36 months	2336	32.1
				37–45 months	30	0.41
BMI	Thin (<18.5)	2080	27.3	Under wt	337	4.6
	Normal (18.5–24.9)	4824	63.4	Normal	5849	80.4
	Over wt./obese (≥25)	708	9.3	Over wt/obese	1086	14.9
Region	Tigray	738	9.7	Tigray	724	10.0
	Afar	550	7.2	Afar	563	7.7
	Amhara	979	12.8	Amhara	924	12.7
	Oromia	1027	13.5	Oromia	989	13.6
	Somali	646	8.5	Somali	642	8.8
	Benishangul	595	7.8	Benishangul	546	7.5
	SNNPR	897	11.8	SNNPR	947	13.0
	Gambella	490	6.4	Gambella	435	6.0
	Harari	475	6.2	Harari	419	5.8
	Addis Ababa	764	10.0	Addis Ababa	610	8.4
	Dire Dawa	473	6.2	Dire Dawa	473	6.5
Mother’s education	No formal education	4,78	62.7	Child sex		
	Primary school (G: 1–8)	1820	23.8			
	High school (G: 9–12)	792	10.4	Male	3888	53.5
	Tertiary (G: >12)	239	3.1	Female	3384	46.5
Mother’s Job	Employed	2193	28.7			
	Per time or irregularly paid	1021	13.4			
	Housewife	3447	45.2			
	Unpaid family worker	973	12.8			
Wealth Index (SES)	Poorest	3024	39.6			
	Middle	1501	19.7			
	Richest	3109	40.7			

BMI: body mass index; SES: socioeconomic status of household.

**Table 3:** Fruits and vegetables consumed by WRA and children within 24HDR.

Frequency or Variety of Any Fruit Consumed p/24HDR						Frequency or Variety of Any Vegetables Consumed p/24HDR					
Children			WRA			Children			WRA		
**Diversity (n) and/or Frequency of Fruits Consumed**	n	%	Diversity (n) and/or Frequency of Fruits Consumed	n	%	Diversity (n) and/or Frequency of Vegetables Consumed	n	%	Diversity (n) and/or Frequency of Vegetables Consumed	n	%
None consumed (0)	6861	94.4	None consumed (0)	5430	65.79	None consumed (0)	2839	39.0	None consumed (0)	1989	24.1
1	300	4.1	1	1045	12.7	1	900	12.4	1	763	9.2
2	77	1.1	2	1039	12.6	2	1278	17.6	2	1587	19.2
≥3	34	0.5	≥3	740	9.0	3	800	11.0	3	1312	15.9
						4	685	9.4	4	1055	12.8
						≥5	770	10.6	≥5	1548	18.8

**Table 4:** Energy and nutrient composition of NOVA foods consumed by WRA.

NOVA Foods Consumed in WRA (n = 8254)					
Nutrient Types	NOVA1	NOVA2	NOVA3	NOVA4	NOVA Total
	p50 (p25, p75, p95)	p50 (p25, p75, p95)	p50 (p25, p75, p95)	p50 (p25, p75, p95)	p50 (p25, p75, p95)
Vit A (µg RAE)	29.9 (4.1, 205.8, 1573.1)	0.0 (0.0, 0.0, 0.0)	0.0 (0.0, 0.0, 6.4)	0 (0.0, 0.0, 0.0)	32.7 (5.3, 207.0, 1576.6)
Thiamin, B1 (mg)	1.2 (0.7, 1.8, 3.1)	0.0 (0.0, 0.0, 0.0)	0.0 (0.0, 0.0, 0.1)	0 (0.0, 0.0, 0.0)	1.2 (0.8, 1.8, 3.2)
Riboflavin, B2 (mg)	0.9 (0.5, 1.4, 2.6)	0.0 (0.0, 0.0, 0.01)	0.0 (0.0, 0.0, 0.3)	0 (0.0, 0.0, 0.0)	0.9 (0.5, 1.4, 2.7)
Niacin, B3 (mg)	7.8 (4.9, 12.1, 22.4)	0.0 (0.0, 0.0, 0.1)	0.0 (0.0, 0.0, 0.6)	0 (0.0, 0.0, 0.0)	8.0 (4.97, 12.4, 23.3)
Vit C (mg)	21.7 (9.8, 46.8, 137.6)	0.0 (0.0, 0.0, 0.0)	0.0 (0.0, 0, 3.5)	0 (0.0, 0.0, 0.0)	22.8 (10.4, 48.1, 139.6)
Calcium (mg)	332.4 (169.9, 598.0, 1473.5)	1.2 (0.4, 2.5, 7.0)	0.0 (0.0, 0.0, 60.2)	0 (0.0, 0.0, 0.0)	347.0 (180.9, 618.6, 1501.8)
Iron (mg)	36.89 (21.42, 63.28, 144.97)	0.02 (0.0, 0.04, 0.1)	0.0 (0.0, 0.0, 4.5)	0 (0.0, 0.0, 0.0)	38.0 (22.2, 65.3, 147.8)
Zinc (mg)	6.4 (3.5, 10.5, 20.5)	0.0 (0.0, 0.0, 0.1)	0.0 (0.0, 0.0, 0.4)	0 (0.0, 0.0, 0.0)	6.6 (3.7, 10.9, 21.3)
Energy (kcal)	1349.8 (912.2, 1979.9, 3294.7)	42.1 (0.0, 185.9, 627.9)	0.0 (0.0, 0.0, 348.6)	0.0 (0.0, 0.0, 263.4)	1587.0 (1095.9, 2254.9, 3621.4)
Tot protein (kcal)	145.9 (93.8, 217.7, 378.1)	0.0 (0.0, 0.0, 0.7)	0.0 (0.0, 0.0, 14.6)	0.0 (0.0, 0.0, 0.0)	150.7 (96.9, 223.9, 387.3)
Tot CHO (kcal)	1069.7 (722.1, 1572.7, 2662.0)	0.0 (0.0, 33.4, 255.2)	0.0 (0.0, 0.0, 50.2)	0.0 (0.0, 0.0, 0.0)	1136.5 (778.2, 1648.2, 2715.6)
Total fat (kcal)	106.5 (60.6, 186.3, 431.7)	0.4 (0.0, 108.5, 503.5)	0.0 (0.0, 0.0, 27.1)	0.0 (0.0, 0.0, 254.8)	211.9 (114.5, 380.3, 854.5)
Phy (mg)	1135.1 (647.4, 1833.0, 3476.5)	0.0 (0.0, 0.0, 0.0)	0 (0.0, 0.0, 0.0)	0 (0.0, 0.0, 0.0)	1190.2 (683.4, 1930.0, 3862.4)
Phy:Fe	2.3 (1.5, 4.2, 7.4)	0.0 (0.0, 0.0, 0.0)	0 (0.0, 0.0, 0.0)	0 (0.0, 0.0, 0.0)	3.1 (1.9, 6.1, 24.2)
Phy:Zn	18.4 (11.8, 29.7, 76.4)	0.0 (0.0, 0.0, 0.0)	0 (0.0, 3.4, 34.6)	0 (0.0, 28.7, 31.5)	18.0 (11.4, 29.1, 72.9)
Phy:Ca	0.2 (0.1, 0.3, 0.9)	0.0 (0.0, 0.0, 0.0)	0 (0.0, 0.0, 0.3)	0 (0.0, 0.3, 0.5)	0.4 (0.2, 0.5, 0.8)
PhyxCa/Zn	142.8 (61.5, 338.2, 1405.6)	0.0 (0.0, 0.0, 0.0)	0 (0.0, 0.3, 38.9)	0 (0.0, 4.3, 88.4)	148.8 (93.0, 272.7, 380.2)
Added salt (g)		6.2 (2.6, 12.6, 30.3)			
Added oil (g)		6.7 (0.0, 26.4, 94.3)			
Added sugar (g)		0.0 (0.0, 18.8, 116.1)			

**Table 5:** Energy and nutrient composition of NOVA foods consumed in children.

NOVA Foods Consumed in Children (n = 7272)					
Nutrient Types	NOVA1	NOVA2	NOVA3	NOVA4	NOVA Total
	p50 (p25, p75, p95)	p50 (p25, p75, p95)	p50 (p25, p75, p95)	p50 (p25, p75, p95)	p50 (p25, p75, p95)
Vit A (µg RAE)	20.8 (1.1, 120.9, 513.1)	0.0 (0.0, 0.0, 6.4)	0.0 (0.0, 0.0, 0.0)	0.0 (0.0, 0.0, 0.0)	24.4 (1.4, 129.0, 528.7)
Thiamin, B_1_ (mg)	0.3 (0.2, 0.6, 1.2)	0.0 (0.0, 0.0, 0.2)	0.0 (0.0, 0.0, 0.0)	0.0 (0.0, 0.0, 0.0)	0.4 (0.2, 0.7, 1.3)
Riboflavin, B_2_ (mg)	0.3 (0.1, 0.6, 1.3)	0.0 (0.0, 0.0, 0.2)	0.0 (0.0, 0.0, 0.0)	0.0 (0.0, 0.0, 0.0)	0.4 (0.2, 0.7, 1.5)
Niacin, B_3_ (mg)	2.4 (1.1, 4.5, 9.4)	0.0 (0.0, 0.0, 1.0)	0.0 (0.0, 0.0, 0.1)	0.0 (0.0, 0.0, 0.2)	2.7 (1.3, 4.9, 10.5)
Vit C (mg)	7.6 (2.6, 17.1, 56.8)	0.0 (0.0, 0.0, 1.6)	0.0 (0.0, 0.0, 0.1)	0.0 (0.0, 0.0, 0.0)	8.0 (2.8, 18.1, 59.6)
Calcium (mg)	134.2 (50.7, 301.5, 776.3)	0.4 (0.0, 1.9, 67.2)	0.0 (0.0, 0.0, 10.0)	0.0 (0.0, 0.0, 4.2)	152.6 (59.9, 334.5, 849.7)
Iron (mg)	9.0 (4.0, 17.6, 43.6)	0.0 (0.0, 0.0, 3.8)	0.0 (0.0, 0.0, 0.6)	0.0 (0.0, 0.0, 0.2)	9.9 (4.4, 19.2, 46.5)
Zinc (mg)	1.8 (0.9, 3.4, 7.6)	0.0 (0.0, 0.0, 0.9)	0.0 (0.0, 0.0, 0.1)	0.0 (0.0, 0.0, 0.1)	2.0 (1.0, 3.7, 8.3)
Energy (kcal)	435.3 (224.4, 742.8, 1395.5)	23.2 (0.0, 112.5, 387.4)	0.0 (0.0, 0.0, 40.7)	0.0 (0.0, 0.0, 147.2)	553.0 (298.0, 912.8, 1648.0)
Tot CHO (kcal)	300.6 (136.9, 546.1, 1077.9)	0.0 (0.0, 43.8, 224.0)	0.0 (0.0, 0.0, 10.5)	0.0 (0.0, 0.0, 40.7)	364.7 (181.8, 619.9, 1164.1)
Tot protein (kcal)	49.3 (24.5, 86.6, 169.0)	0.0 (0.0, 0.0, 20.4)	0.0 (0.0, 0.0, 2.8)	0.0 (0.0, 0.0, 3.1)	54.7 (27.8, 93.4, 179.0)
Total Fat (kcal)	51.5 (20.2, 114.0, 298.3)	0.0 (0.0, 38.8, 217.4)	0.0 (0.0, 0.0, 3.9)	0.0 (0.0, 0.0, 94.9)	96.2 (40.7, 200.5, 474.1)
Phy (mg)	283.6 (96.4, 584.0, 1322.9)	0.0 (0.0, 0.0, 115.1)	0.0 (0.0, 0.0, 0.0)	0.0 (0.0, 0.0, 17.4)	313.1 (116.4, 625.9, 1414.1)
Phy:Fe	2.2 (1.4, 4.0, 7.9)	0.0 (0.0, 0.0, 4.2)	0.8 (0.0, 2.4, 13.5)	14.1 (4.2, 14.1, 15.6)	2.3 (1.4, 4.0, 7.6)
Phy:Zn	15.3 (7.3, 25.8, 62.6)	0.0 (0, 0.0, 0.0, 33.0)	0.0 (0.0, 0.0, 0.0)	0.0 (0.0, 0.0, 28.7)	15.3 (7.6, 25.3,59.8)
Phy:Ca	0.2 (0.1, 0.3, 0.8)	0.0 (0.0, 0.0, 0.5)	0.1 (0.0, 0.2, 1.0)	0.3 (0.3, 0.3, 0.5)	0.6 (0.3, 0.9, 1.4)
PhyxCa/Zn	40.5 (11.8, 109.7, 417.7)	0.0 (0.0, 0.0, 14.2)	1.0 (0.0, 15.9, 274.0)	3.2 (1.8, 8.9, 27.1)	59.4 (32.1, 106.5, 171.9)
Added Salt (g)		0.8 (0.0, 3.5, 57.9)			
Added Oil (g)		0.0 (0.0, 5.1, 41.2)			
Added Sugar (g)		0.0 (0.0, 2.3, 52.6)			

**Table 6:** Calorie, recommended nutrient intake (RNI), and percent achieved.

	Subjects by Age Category											
	Children, 6–12 Months Old (n = 1804)			Children, 1–4 Years Old (n = 5468)			Women (15–18) Years Old (n = 388)			Women, 19+ Years Old (n = 7866)		
Nutrients and Energy	Intake, Median (IQR)	RNI	% NRI Achieved	Intake, Median (IQR)	RNI	% NRI Achieved	Intake, Median (IQR)	RNI	% NRI Achieved	Intake, Median (IQR)	RNI	% NRI Achieved
Energy (Kcal)	282.4 (358.3)	872.5	32.4	654.5 (621.1)	1230	53.2	1523.8 (1110.4)	2200	69.3	1586.2 (1158.4)	2200	72.1
CHO (g)	38.2 (59.3)	95	40.2	110.7 (110.1)	130	84.7	271.9 (211.5)	130	209.2	284.8 (217.7)	130	219.1
Protein (g)	7.0 (10.5)	11.0	63.6	16.0 (16.7)	13.0	123.1	39.6 (32.3)	46.0	86.1	37.6 (31.6)	46.0	81.7
Fat (kcal)	72.6 (140)	30%E	85.7	104.0 (163.8)	30%E	53.0	276.8 (312)	30%E	60.6	208.9 (260.4)	30%E	43.9
Fe (mg)	4.0 (7.0)	9.3	43.0	12.5 (15.7)	5.8	215.5	44.3 (48.7)	31.0	142.9	37.8 (42.8)	29.4	128.6
Ca (mg)	118.3 (282.5)	400	29.6	164.3 (278.8)	500	32.9	361.4 (409.3)	1000	36.1	346.5 (439.6)	1000	34.7
Zn (mg)	1.0 (1.4)	8.4	11.9	2.5 (3.0)	8.3	30.1	7.5 (7.6)	9.8	76.5	6.6 (7.2)	9.8	67.4
Vit A (µg RAE)	31.7 (107.9)	400	7.9	23.2 (140.1)	400	5.8	46.1 (213.0)	600	7.7	31.8 (201.2)	500	6.4
Thiamin (mg)	0.2 (0.2)	0.3	66.7	0.5 (0.5)	0.5	100	1.2 (1.0)	1.1	109.1	1.2 (1.0)	1.1	109.1
Riboflavin (mg)	0.2 (0.4)	0.4	50	0.4 (0.5)	0.5	80	0.9 (0.9)	1.0	90	0.9 (0.9)	1.1	90
Niacin (mg NE)	1.3 (2.1)	4.0	32.5	3.3 (3.8)	6.0	55	8.0 (7.5)	16.0	50	8.0 (7.4)	14.0	57.1
Vit C (mg)	4.4 (10.0)	30	14.7	9.7 (17.5)	30	32.3	28.1 (40.6)	40	70.3	22.3 (37.4)	45	49.6

IQR: Interquartile range; CHO: Carbohydrate; E: Energy; kcal: kilocalorie; µgRAE: Micrograms of retinol activity equivalents; mgNE: milligrams of niacin equivalents; iron (Fe); RNI: recommended nutrient intake; 10% bioavailability for iron and low bioavailability for zinc RNI was used. The % achieved was determined using nutrient RNI, adapted from [33,34,37,38,43] for minerals and vitamins, whereas for energy in children, [36] was used, and for fat [43] and RNI for Protein, carbohydrates, and particularly energy for adults using the Institute of Medicine (IOM) thresholds [35].

**Table 7:** Mean UPF (kcal) intake in children and WRA by region.

Regions	Children				WRA			
	UPF (kcal)	95% CI	Total Energy (kcal)	% UPF kcal	UPF (kcal)	95% CI	Total Energy (kcal)	% UPF kcal
Tigray (n = 812)	22.8	17.5, 28.1	749.8	3.0	32.0	26.0, 38.0	2066.9	1.6
Afar (n = 617)	12.2	7.6, 16.8	713.8	1.7	14.3	7.4, 21.1	2210.9	0.7
Amhara (n = 1077)	21.1	16.3, 26.0	664.6	3.2	33.8	27.8, 39.9	2085.5	1.6
Oromia (n = 1093)	28.8	22.9, 34.6	715.2	4.0	33.0	24.6, 41.3	1860.3	1.8
Somali (n = 687)	23.9	17.0, 30.8	601.8	4.0	17.2	10.5, 24.0	1338.0	1.3
Benishangul (n = 629)	18.0	12.2, 23.8	633.9	2.8	22.2	17.3, 27.1	1812.3	1.2
SNNPR (n = 1049)	9.8	7.3, 12.2	543.7	1.8	20.6	15.5, 25.8	1610.7	1.3
Gambela (n = 511)	12.7	6.4, 19.1	569.6	2.2	1.5	0.2, 2.9	1557.1	0.1
Harari (n = 488)	50.5	37.3, 63.7	775.8	6.5	53.9	40.3, 67.5	1799.4	3.0
Addis Ababa (n = 802)	63.4	54.2, 72.6	750.6	8.5	186.3	166.3, 206.3	1554.0	12.0
Dire Dawa (n = 489)	35.5	26.6, 44.5	687.9	5.2	32.5	21.5, 43.5	1441.1	2.3
National	24.8	22.9, 26.8	669.6	3.7	42.0	22.9, 26.8	1780.4	2.4

For region-based UPF intake, we reported the mean, as the median was extremely low because of large energy data variability.

**Table 8:** Sociodemographic factors with UPF consumption in children.

Predictor Variables	n	%	Daily Kcal Intake from UPF in Children			
			Exp (β) [95% CI]	SE	*p*-Value	Q-Value
Residence						
Urban	1945	26.75	2.55 [1.67, 3.87]	0.54	0.000 *	0.000
Rural	5327	73.25	1			
Child age						
6–12 months	1804	24.81	1			
13–24 months	3102	42.66	1.27 [1.14, 1.41]	0.07	0.000 *	0.000
25–36 months	2336	32.12	1.58 [1.41, 1.77]	0.09	0.000 *	0.000
37–45 months	30	0.41	6.75 [3.36,13.55]	5.37	0.000*	0.000
BMI z-score						
Under Wt	337	4.63	1			
Normal	5849	80.43	1.13 [0.98, 1.31]	0.08	0.10	0.10
Over Wt	1086	14.93	1.11 [0.86, 1.44]	0.15	0.405	0.405
SES						
Poorest	3187	43.83	1			
Middle	1438	19.77	1.17 [1.03, 1.32]	0.07	0.016	0.01
Richest	2647	36.40	1.23 [1.05, 1.44]	0.10	0.010	0.008
Mother’s Education						
No formal education	4675	64.29	1			
Primary school (G: 1–8)	1683	23.14	1.51 [1.35, 1.70]	0.09	0.000 *	0.000
High school (G: 9–12)	717	9.86	1.77 [1.50, 2.10]	0.15	0.000 *	0.000
Tertiary (G: >12)	197	2.78	1.47 [1.12, 1.93]	0.20	0.005 *	0.006
Mother job						
Employed	1946	26.76	1			
Per-time/irregularly paid	1082	14.88	0.99 [0.86, 1.15]	0.07	0.918	
Housewife	3730	51.29	0.85 [0.76, 0.96]	0.05	0.007 *	0.0071
Other (family supporter)	514	7.07	1.01 [0.84, 1.22]	0.10	0.894	
_cons			4.85 [3.37, 6.98]	0.90	0.000	
/logs			0.42 [0.40, 0.43]	0.01		
Random effect						
Region var(_cons)			0.14 [0.04, 0.51]	0.09		
Region > cluster var(_cons)			1.59 [1.34, 1.88]	0.14		

BMI z-score: BMI-for-age z-score; * *p*-value significant if *p* ≤ Q (Holm–Bonferroni adjusted value).

**Table 9:** Determinants of less-processed (NOVA1) food intake in children.

Predictor Variables	n	%	Children’s Daily Kcal Intake from Less-Processed (NOVA1) Foods			
			Exp (β) [95% CI]	S.E	*p*-Value	Q-Value
Residence				0.05	0.617	
Urban	1945	26.75	1.02 [0.94, 1.12]			
Rural	5327	73.25	1			
Child age						
6–12 months	1804	24.81	1			
13–24 months	3102	42.66	1.78 [1.70, 1.86]	0.04	0.000 *	0.000
25–36 months	2336	32.12	2.43 [2.32, 2.55]	0.06	0.000 *	0.000
37–45 months	30	0.41	2.16 [1.64, 2.85]	0.30	0.000 *	0.000
BMI Z-score						
Under Wt	337	4.63	1.07 [1.00, 1.14]	0.05	0.035	0.01
Normal	5849	80.43	1.15 [1.03, 1.29]	0.07	0.012	0.008
Over Wt	1086	14.93	1			
SES						
Poorest	3187	43.83	1.23 [1.16, 1.31]	0.04	0.000 *	0.000
Middle	1438	19.77	1.14 [1.07, 1.21]	0.04	0.000 *	0.000
Richest	2647	36.40	1			
Mother’s Education						
No formal education	4675	64.29	1			
Primary school (G: 1–8)	1683	23.14	1.00 [0.96, 1.05]	0.02	0.997	
High school (G: 9–12)	717	9.86	1.16 [1.08, 1.25]	0.04	0.000 *	0.000
Tertiary (>12)	197	2.71	1.29 [1.15, 1.45]	0.08	0.000 *	0.000
Mother job						
Employed (formal)	1946	26.76	1			
Per-time or irregularly paid	1082	14.88	1.01 [0.96, 1.08]	0.03	0.626	
Housewife	3730	51.29	0.99 [0.94, 1.03]	0.02	0.539	
Others (unpaid family supporter)	514	7.07	1.36 [1.26, 1.47]	0.05	0.000 *	0.000
_cons			238.38 (221.60, 79.42)	14.69	0.000	
/logs			−0.31 (−0.33, −0.29)	0.01		
Random effect						
Region var(_cons)			0.02 (0.007, 0.05)	0.009		
Region > cluster var(_cons)			0.04 (0.03, 0.05)	0.005		

BMI Z-score: BMI-for-age z-score * *p*-value significant if *p* ≤ Q (Holm–Bonferroni adjusted value).

**Table 10:** Determinants of UPF consumption in WRA.

Variables	n	(%)	Kcal Intake from UPF in WRA			
			Exp (β) [95% CI]	SE	*p*-Value	Q-Value
Residence				0.87	0.000 *	0.000
Urban	2142	28.06	3.77 [2.40, 5.92]			
Rural	5492	71.94	1			
Mother’s age (years)						
Teen/young adult (15–21)	1296	16.98	1.03 [0.91, 1.18]	0.07	0.626	
Early adulthood (22–34)	4982	65.26	1.12 [1.01, 1.23]	0.06	0.029	0.007
Middle age (35–45)	1356	17.76	1			
Mother BMI						
Thin (BMI < 18.5)	2080	27.33	1			
Normal (BMI 18.5–24.9)	4824	63.37	1.05 [0.96, 1.1.15]	0.05	2.73	2.73
OverWt/obese (BMI ≥ 25)	708	9.30	1.23 [1.06, 1.44]	0.10	0.008	0.006
SES						
Poorest	3024	39.61	1			
Middle	1501	19.66	0.98 [0.88, 1.10]	0.06	0.770	0.770
Richest	3109	40.73	1.39 [1.20, 1.63]	0.11	0.000 *	0.000
Mother Education						
No formal Education	4783	62.65	1			
Primary (G: 1–8)	1820	23.84	1.38 [1.24, 1.53]	0.07	0.0008 *	0.005
High school (G: 9–12)	792	10.37	1.66 [1.42, 1.94]	0.13	0.000 *	0.000
Tertiary (G: >12)	239	3.13	1.41 [1.12, 1.78]	0.16	0.003 *	0.005
Mother job						
Employed	2193	28.73	1			
Per-time or irregularly paid	1021	13.37	0.94 [0.82, 1.08]	0.07	0.380	0.380
Housewife	3447	45.15	0.97 [0.88, 1.08]	0.05	0.610	0.610
Unpaid family worker	973	12.75	1.01 [0.87, 1.18]	0.08	0.898	0.898
_cons			4.24 [2.71, 6.65]	0.97	0.000	
/logs			0.37 [0.35, 0.38]	0.01		
Random effect						
Region var(cons)			0.50 [0.20, 1.29]	0.24		
Region > cluster var(cons)			2.03 [1.72, 2.39]	0.17		

* *p*-value significant if *p* ≤ Q (Holm–Bonferroni adjusted value).

**Table 11:** Factors associated with energy intake from NOVA1 in WRA.

Variables	n	%	Kcal Intake from Less-Processed (NOVA1) Foods in WRA			
			Exp (β) [95% CI]	SE	*p*-Value	Q-Value
Residence				0.03	0.000 *	0.000
Urban	2142	28.06	0.80 [0.74, 0.86]			
Rural	5492	71.9	1			
Mother Age						
Teen/young adult (15–21)	1296	16.98	1.06 [1.02, 1.11]	0.02	0.003 *	0.004
Early adulthood (22–34)	4982	65.26	1.03 [1.00, 1.07]	0.02	0.034	0.005
Middle age (35–45)	1356	17.76	1			
BMI Mother						
Thin (BMI < 18.5)	2080	27.33	1			
Normal (BMI 18.5–24.9)	4824	63.37	1.01 [0.98, 1.04]	0.01	0.563	0.563
Overwt/obese (BMI ≥ 25)	708	9.30	1.02 [0.97, 1.07]	0.03	0.534	0.534
SES						
Poorest	3024	39.61	1			
Middle	1501	19.66	1.02 [0.98, 1.05]	0.02	0.371	0.371
Richest	3109	40.73	1.02 [0.98, 1.06]	0.02	0.390	0.390
Mother Education						
No formal education	4783	62.65	1			
Elementary school (G: 1–8)	1820	23.84	1.00 [0.97, 1.04]	0.02	0.792	0.792
High school (G: 9–12)	792	10.37	1.03 [0.98, 1.08]	0.03	0.236	0.236
Tertiary (>12)	239	3.13	1.06 [0.99, 1.14]	0.04	0.104	0.104
Mother job						
Employed	2193	28.73	1			
Per-time or irregularly paid	1021	13.37	1.02 [0.98, 1.07]	0.02	0.297	0.297
Housewife	3447	45.15	0.96 [0.93, 0.99]	0.02	0.016	0.005
Unpaid family worker	973	12.75	0.98 [0.94, 1.03]	0.02	0.460	0.460
_cons			1417.6 [1283.0, 1566.3]	72.18	0.000	
/logs			−0.69 [−0.71, −0.68]	0.01		
Random effect						
Region var(_cons)			0.02 [0.01, 0.05]	0.01		
Region > cluster var(_cons)			0.03 [0.03, 0.04]	0.00		

* *p*-value significant if *p* ≤ Q (Holm–Bonferroni adjusted value).

## Data Availability

The data supporting the conclusions of this article will be made available by the authors upon request.

## Supplementary Materials

The following supporting information can be downloaded at: https://www.mdpi.com/article/10.3390/nu17172818/s1, Lists of ingredients and energy intake from each NOVA by residences in WRA and children.
