# Supplementary material for: Energy and Nutrient Intake Gaps and Socioeconomic Determinants of Ultra-Processed and Less-Processed Foods Consumed in Ethiopia: Evidence from National Food Consumption Survey
Source: Nutrients. 2025 Aug 29;17(17):2818. doi: 10.3390/nu17172818 (PMC12430034; doi:10.3390/nu17172818)
Supplement: Supplementary file 1 [file nutrients-17-02818-s001.zip › Suplementary TableS1, TableS2 and TableS3_Nutrients.pdf]

The food items and ingredients were classified in the NOVA system by categorizing into four groups: Group 1: ‘unprocessed or minimally processed foods’ (e.g. fresh, dry or frozen grains such as rice, meat, fish, cereal-based foods, roots and tubers, natural spices, and herbs, fruits, and vegetables); Group 2: ‘processed culinary ingredients’ (such as. edible oils, sauce, sugar, salt), extracted from foods or from nature and used in kitchens to make culinary preparations); Group 3: ‘processed foods’ (foods manufactured with the addition of salt, sugar or other processed culinary ingredients to unprocessed or minimally processed foods, such as salted, boiled wheat noodles), alcoholic fermented beverages (Tella, Tejj, Birz), baby food cereal, fafa porridge; skimmed milk, bouillon powder, sardine canned oil; and Group 4: ‘ultra-processed foods’ (e.g. soft drinks either packed with plastic or glass bottles, salty snacks, confectionery, ready-to-heat frozen meals, sugary juices, ice pops, confectionery, cake, cookies, biscuits, chips, samosa, processed baby foods, high alcoholic drinks such as gin, whisky.

Supplemental Table S1. Lists of consumed NOVA-based ingredients

NOVA 1	NOVA 2	NOVA 3	NOVA 4
**Cereal based:** Chickpeas, pea roasted, wheat bread, chickpeas shiro wot (stew), (tef+sorghum: Enjera), rice white unenriched, sorghum enjera, peas field split, atmit (tef & sorghum flour+birds eye chili: unfermented), lentil boiled, besso (wheat powder paste with water & salt/sugar), potato stew, wheat unleavened bread, barley enjera, wheat+maize roasted, lentil split, wheat white porridge, wheat+maize (unleavened bread), sugar cane, pea shiro stew, sorghum+fish: steamed (porridge), linseed flour, com whole boiled (chickpea+pea powder stew), linseed dried, (tef+sorghum: enjera), millet white enjera, millet unleavened bread, lentil sauce without chilli, barley white whole roasted, wheat white whole roasted, sorghum white whole grain, wheat white split grain, peas field whole dried, maize bread (maize+wheat: unleavened bread), com yellow whole roasted, kidney beans whole boiled, barley+oat flour mix (porridge), safflower seed roasted, niger seed boiled, lupine boiled, peanut butter, safflower seed roasted, pasta white cooked, rice brown cooked.	Sugar (brown), white sugar, plain salt, soybean oil, peanut oil, olive oil, corn and canola oil, cooking oil, butter, fish oil, high oleic oil, cod liver oil, niger seed oil, sesame seed oil, safflower oil, butter oil (ghee cow), honey, butter without	Kellogg's corn flakes; beef+(tomato, potato, chili, fenugreek, shallot, garlic, salt); (Tella, Tejj, Birz) (local alcoholic fermented beverages); shamita (local alcoholic beverage with barely); baby food cereal; Pea flour & (shallot + oil + salt and spices); splited broad bean & (pea flour + spices + butter + salt); tomato paste (canned); milk cow & (coffee husk + sugar + salt + water: beverage); beer commercial (4% alcohol); alcoholic beverage; emmer wheat & (sugar + salt + oil: soup), potato & (shallot + oil + spices + salt: sauce), kale with (shallot + oil + salt + spices: sauce); fafa porridge; skimmed milk; splited broad bean & (pea flour + shallot + spices + butter + salt); table wine (11.5%); grass pea & broad bean flour (1:1) with (oil + shallot + salt: sauce); cheese soft fresh low fat (cottage); kale & (butter + spices + salt); cheese soft fresh (ricotta); wheat flour & (butter +	Palm oil, shortenings, ghee, magarine, shenolega, soft drinks packed with plastic & glass bottles, sugary juices, ice pops, confectionery, cake, cookies, biscuits, chips, samosa, processed baby foods, high alcohol drinks
**Fruits & Vegetables**: Prickly pear fresh fruit, peach fresh, tomato raw, shallot raw, green pepper, banana, kale raw, orange fresh, tomato fried, tomato boiled, potato Irish, cabbage boiled, prickly pear, carrot raw, date fresh, chili raw, lemon fresh,			

mango fresh, cabbage raw, swiss chard raw, swiss chard boiled, melons cantaloupe raw, carrot boiled, tomato red boiled, avocado fresh, papaya fresh, beets cooked, squash boiled, mushrooms (stem and cap), false banana (enset) bulla, raddish raw, pineapple fresh, green onion fresh, apricots raw, pears raw, spinach raw, citron fresh, moringa stenopetala boiled, bread fruit ripe, apple fresh, grapes fresh, cauliflower raw, chili (unspiced), guava fresh, cucumber raw, lettuce raw.	salt, leavening agents, fresh bouillon powder (Ersho), yeast compressed anhydrous, baking powder, curry powder, NaAl(SO4)2, starch raw, butter spiced, butter-milk, butter+birds eye camel milk with butter & chili	salt: unleavened bread); (wheat flour + sugar, water, milk, oil: soup); (splited broad bean & lentil (3:1) + oil + shallot + salt: sauce); milk cow evaporated whole canned unsweetened; bouillon powder; (broad bean & chickpea flour + oil + shallot + salt: sauce); sardine canned oil; splited broad bean + (shallot + oil + spices + salt: sauce), chilli + shallot + salt + oil: sauce); false banana (refined), (butter + sugar + salt: gruel); teff & (oil + beberre + salt + water + fafa: porridge); Agenbegne (unidentified vegetable) with (shallot + oil + spices + salt: sauce); peanut butter with agenbegne + salt: sauce); kidney bean & (okra powder + shallot + oil + salt: sauce); sorghum & (maize + mushroom + shallot + garlic + chilli + butter + salt: sauce); cottage cheese & (toasted kale + butter + chilli + spices & salt), wheat flour (refined); sesame seed & (eggs + sugar + salt + baking powder + vanilla (fried); false banana (refined) & (butter + chili + salt: porridge); wheat flour & (oil + sugar + salt: unleavened bread); wheat (refined) & (eggs + shallot + spices + oil + salt: fried); milk powder; fafa porridge/genfo.	(gin & whiskey) & mayonnaise.<br><br>Overall, palm oil was dominantly consumed which took the greatest share of UPF.
**Roots and tubers:** Ginger root raw, garlic fresh, cassava raw, bamboo shoots raw, yam boiled, bamboo shoot+okra powder, taro boiled, taro raw, potato boiled & baked (with + without skin), green pepper boiled, sweet potato raw.			
**Spices and Herbs:** Turmeric, chili spiced, cloves dried, mekmeqo dried, garlic raw, cumin black, fenugreek (white variety), sacred basil dried, cumin fresh dried, cardamom fresh dried, coriander dried, fenugreek stew, spearmint dried, garlic boiled, sacred basil fresh, dat'a (made from papper, ginger, garlic, water and salt), jute potherb leaf, nutmeg ground, mustard green seed raw, cinnamon fresh dried, rue fresh.			
**Beverages:** Tea leaves dried, coffee beans roasted dried.			
**Meat & meat products:** Mutton grilled, mutton raw, beef raw, meat stew, goat meat raw, chicken whole raw, tongue goat, beef liver fried, fish liver raw, fish soup, sheep kidney raw , chicken whole boiled, meat soup, chicken meat stew, pork fresh raw, tuna yellow fin, fish whole dried, river fish dried boiled, sea fish grilled, lake fish dried raw, river fish grilled, leek boiled, tripe beef raw, catfish cooked, sea fish raw, veal raw, ham raw, beef fried.			
**Milk & milk products:** Milk cow fresh, human milk, milk fat whey, camel milk dry W/ vitamin D, milk cow skimmed, hoja (milk, salt, coffee husk, water), yogurt whole cow.			
**Egg and its products:** Egg whole raw, egg, whole boiled.			

Supplement Table S2. Number of the clusters (EAs), households, and participants included in the survey

	WRA			Children		
	**Urban**	**Rural**	**Total**	**Urban**	**Rural**	**Total**
EA (cluster)	88	229	317	88	229	317
HHs	2,251	6,003	8,254	1,945	5,327	7,272
WRA	2,251	6,003	8,254	1,945	5,327	7272

Supplementary Table S3. Energy (kcal) share of NOVA foods in WRA and children.

	Children (n=7272)		WRA (n=8253)	
NOVA energy share (%)	Mean	[95% CI]	Mean	[95% CI]
NOVA1	74.6	[74.2, 75.1]	78.98	[78.6, 79.4]
NOVA2	18.3	[17.9, 18.8]	14.0	[13.68, 14.4]
NOVA3	1.9	[1.7, 2.1]	3.5	[3.3, 3.8]
NOVA4	5.1	[4.9, 5.4]	3.5	[3.3, 3.7]

Energy data did not follow a Gaussian distribution, also since NOVA2, NOVA 3, and NOVA 4 comparably consumed less, in certain cases, they contained zero values; therefore square root transformed energy share data presented for each NOVA food group consumed.
