# Supplementary material for: Energy and Nutrient Intake Gaps and Socioeconomic Determinants of Ultra-Processed and Less-Processed Foods Consumed in Ethiopia: Evidence from National Food Consumption Survey
Source: Nutrients. 2025 Aug 29;17(17):2818. doi: 10.3390/nu17172818 (PMC12430034; doi:10.3390/nu17172818)

Supplementary materials

Figure 1 and Figure 2 below show the overview of energy (kcal) consumed from each NOVA and the sum of the NOVA food groups by urban and rural residents in WRA and children. The amount of energy consumed from NOVA 1 was higher in rural settings than in urban in WRA and children. Total energy consumption was slightly larger for women living in rural settings than in urban, but the opposite for children (Supplement S1 and Supplement S2).

Supplement Figure S1. Median energy consumed from NOVA food in rural and urban residences in WRA

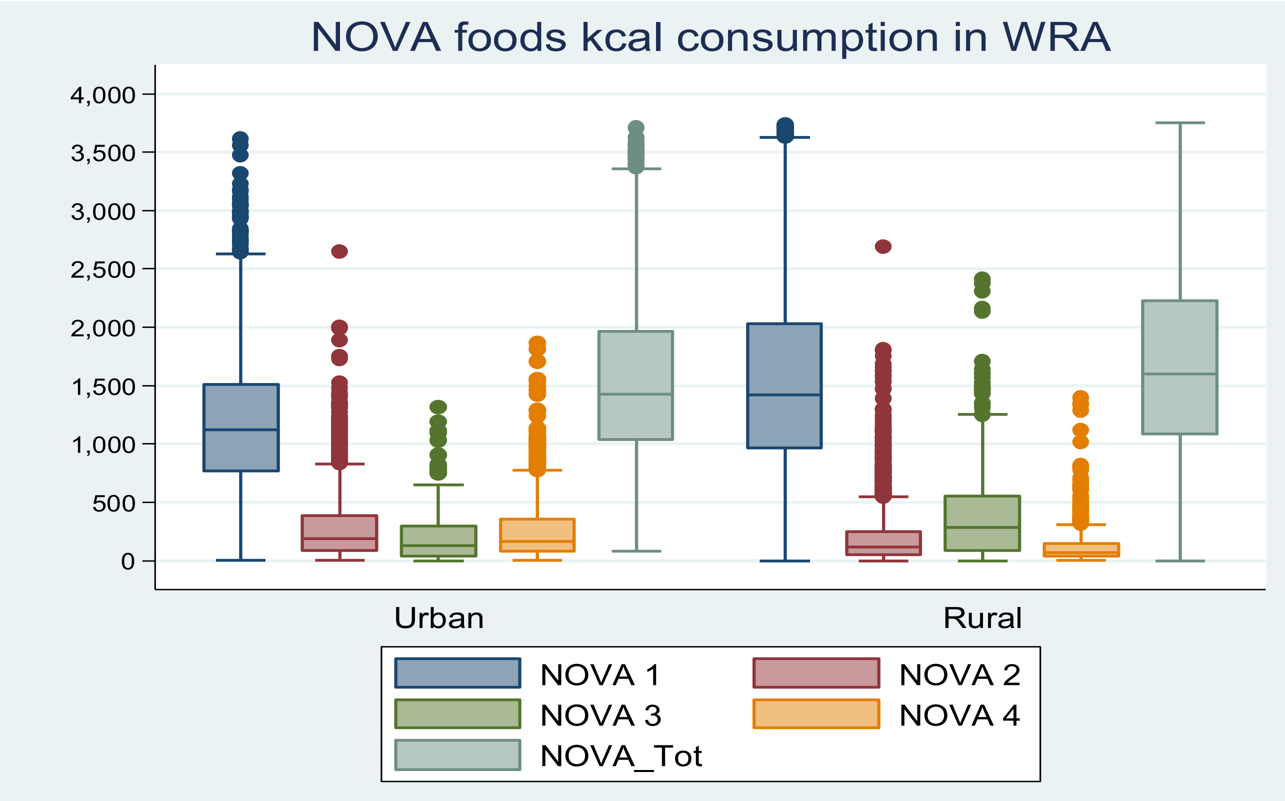

Supplement Figure S2. Median energy consumed by NOVA groups by children in rural and urban residence

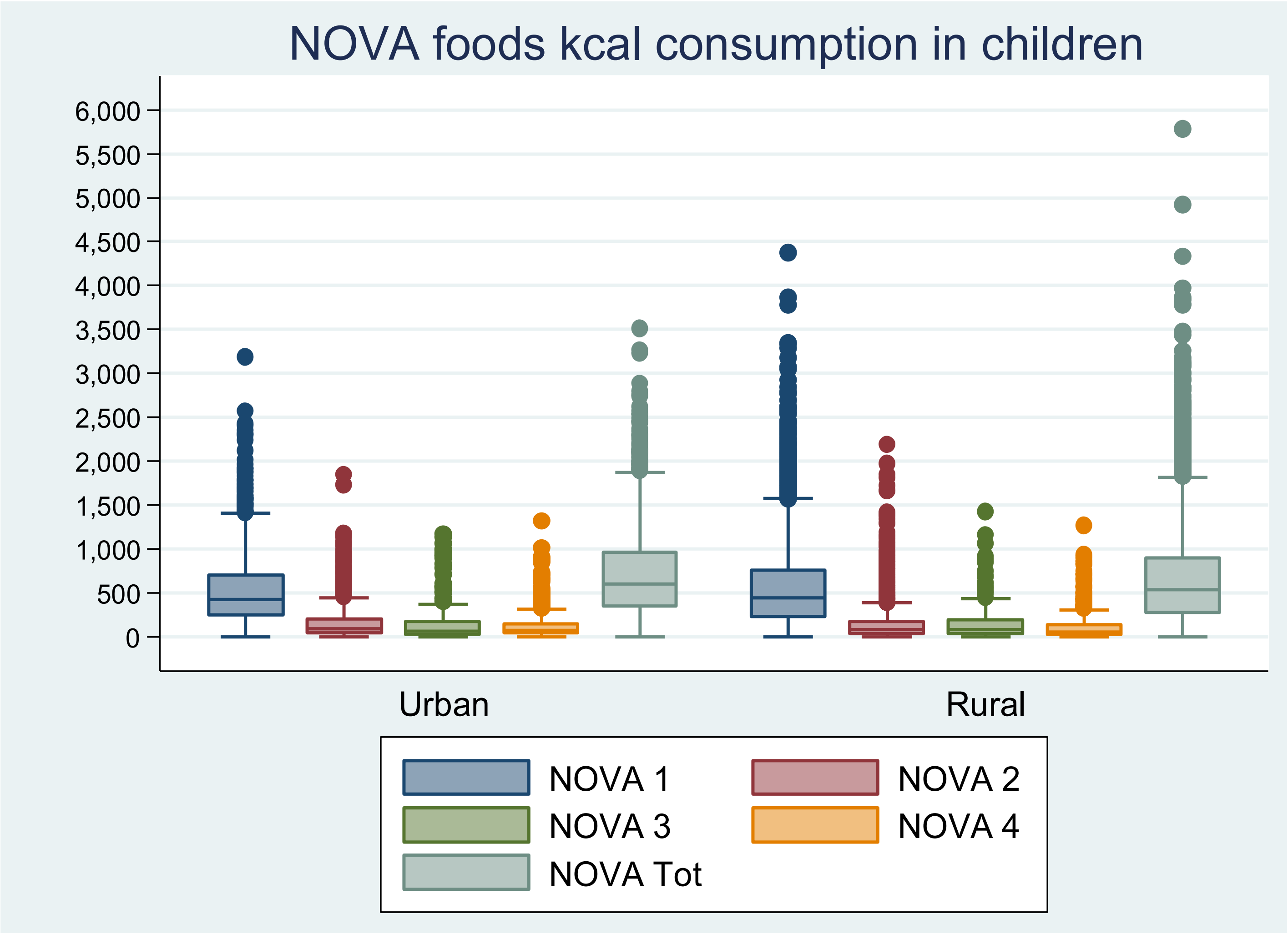

Supplement: Supplementary file 1 [file nutrients-17-02818-s001.zip › Supplementary FigureS1 and FigureS2_Nutrients.pdf]
